# Risk of Stroke in Systemic Necrotizing Vasculitis: A Nationwide Study Using the National Claims Database

**DOI:** 10.3389/fimmu.2021.629902

**Published:** 2021-03-31

**Authors:** Sung Soo Ahn, Minkyung Han, Juyoung Yoo, Yong-Beom Park, Inkyung Jung, Sang-Won Lee

**Affiliations:** ^1^Department of Internal Medicine, Yongin Severance Hospital, Yonsei University College of Medicine, Yongin, South Korea; ^2^Biostatistics Collaboration Unit, Department of Biomedical Systems Informatics, Yonsei University College of Medicine, Seoul, South Korea; ^3^Division of Rheumatology, Department of Internal Medicine, Yonsei University College of Medicine, Seoul, South Korea; ^4^Institute for Immunology and Immunological Diseases, Yonsei University College of Medicine, Seoul, South Korea; ^5^Division of Biostatistics, Department of Biomedical Systems Informatics, Yonsei University College of Medicine, Seoul, South Korea

**Keywords:** systemic necrotizing vasculitis, anti-neutrophil cytoplasmic antibody-associated vasculitis, polyarteritis nodosa, stroke, incidence, microscopic polyangiitis

## Abstract

**Objective:**

Evidences indicate that the risk of stroke is increased in autoimmune rheumatic diseases. This study aimed to investigate the incidence of stroke in patients with systemic necrotizing vasculitis (SNV) using the national health database.

**Methods:**

Data were obtained from the Korean National Claims database between 2010 and 2018 to identify incident SNV [anti-neutrophil cytoplasmic antibody-associated vasculitis (AAV) and polyarteritis nodosa (PAN)] cases. The standardized incidence ratio (SIR) and incidence rate ratio (IRR) were calculated to estimate the risk of stroke in patients with SNV compared to the general population and among disease subgroups. Time-dependent Cox’s regression analysis was performed to identify risk factors for stroke.

**Results:**

Among 2644 incident SNV cases, 159 patients (6.0%) were affected by stroke. The overall risk of stroke was significantly higher in patients with SNV compared to the general population (SIR 8.42). Stroke event rates were the highest within the first year of SNV diagnosis (67.3%). Among disease subgroups, patients with microscopic polyangiitis (MPA) exhibited higher IRR compared to PAN (adjusted IRR 1.98). In Cox’s hazard analysis, older age and MPA were associated with higher risk of stroke [hazard ratio (HR) 1.05 and 1.88], whereas the administration of cyclophosphamide, azathioprine/mizoribine, methotrexate, and statins were protective in stroke (HR 0.26, 0.34, 0.49, and 0.50, respectively).

**Conclusion:**

A considerable number of SNV patients experienced stroke, especially in the early phase of disease. Older age and MPA diagnosis were associated with elevated risk of stroke, while the administration of immunosuppressive agents and statins was beneficial in preventing stroke.

## Introduction

Anti-neutrophil cytoplasmic antibody (ANCA)-associated vasculitis (AAV) is a rare systemic inflammatory disorder causing necrotizing organ injury within small vessels, which is typically associated with myeloperoxidase-ANCA or proteinase 3-ANCA (1). AAV is divided into three distinct diseases according to the clinical, laboratory, and histological characteristics: microscopic polyangiitis (MPA), granulomatosis with polyangiitis (GPA), and eosinophilic granulomatosis with polyangiitis (EGPA) (2, 3). AAV can affect any organ of the body and various clinical symptoms occur depending on the organs affected. On the other hand, polyarteritis nodosa (PAN), which is classified as systemic vasculitis involving the medium-sized vessels, also causes necrotizing vasculitis (4). Owing to the pathologic similarities, AAV and PAN are traditionally considered to comprise a group of systemic necrotizing vasculitis (SNV) (5).

Stroke is defined as a medical condition of acute and focal functional impairment of the brain, retina, or spinal cord; it is associated with significant mortality and disability (6, 7). Stroke is generally divided into hemorrhagic and ischemic subtypes; ischemic stroke is the most common type, accounting for up to 70% of cases (8, 9). Several common risk factors for the occurrence of stroke, such as older age, sex, hypertension, dyslipidemia, diabetes mellitus, atrial fibrillation, and smoking, have been suggested (9, 10). Besides, a growing body of evidence has suggested that chronic inflammation and high degree of inflammation are crucial factors associated with increased vascular thrombosis in autoimmune rheumatic diseases by triggering the coagulation cascade (11, 12). In this context, previous studies have reported an increased risk of stroke in patients with large vessel vasculitis, such as Takayasu arteritis (TA) and giant cell arteritis (GCA). A retrospective study by Hwang et al. demonstrated that more than 10% of patients with TA experienced ischemic stroke (13), and Nesher et al. showed that cranial ischemic complications are common in patients with GCA, implying that patients with systemic vasculitis are at a high risk of developing stroke (14). Mechanistically, the development of atherosclerotic lesions is considered an important cause of stroke (15). Similarly, in the pathogenesis of SNV, subclinical atherosclerosis is accelerated as a consequence of systemic and localized inflammation and deterioration of helper T (Th) cell balance (16). Based on these findings, even though it is possible that the risk of stroke is increased in patients with SNV, the incidence of stroke in patients with SNV has not been investigated in detail. Therefore, this study was conducted to investigate the incidence of stroke in SNV using a nationwide database.

## Materials and Methods

### Data Extraction From the Health Insurance and Review Agency Database

The principal diagnosis and comorbidities [based on International Classification of Diseases (ICD)-10 codes], clinical information (age, sex, geographic area), and the administered medication of the patients were obtained from the Health Insurance and Review Agency (HIRA) database. The HIRA database is a nationwide claims data repository that includes information of medical service utilization of an individual, which is comprised of: general and providers’ information, the use of healthcare services, diagnosis, and the data of drug prescription (17). This information is submitted by medical institutions to the Korean government in order to request monetary reimbursement and is integrated and recorded in the HIRA database. A schematic figure depicting the generation and utilization of HIRA data is described in **Supplementary Figure 1**. By using this database, it is possible to access the information of the entire population (nearly 50 million patients) that is enrolled in the National Health Insurance System of Korea (18).

To identify SNV patients, the respective ICD-10 codes for MPA (M31.7), GPA (M31.3), EGPA (M30.1), and PAN (M30.0) were used. Patients were diagnosed with SNV when they were first registered with the corresponding ICD-10 codes for AAV or PAN in a general or tertiary hospital and were prescribed with glucocorticoids (betamethasone, dexamethasone, methylprednisolone, prednisone, prednisolone, hydrocortisone, triamcinolone, budesonide, and deflazacort) during the follow-up period (19). The date on which the diagnosis of SNV was first registered in the HIRA database was defined as the index date, and the medications of immunosuppressive agents (glucocorticoids, cyclophosphamide, rituximab, azathioprine/mizoribine, and methotrexate), antiplatelet agents (aspirin, clopidogrel), and statins that were prescribed after the diagnosis of SNV were also counted.

The entire data of the study population were first extracted from the HIRA database between January 2008 and December 2018, and a 2-year washout period was given to exclude patients with the diagnosis of SNV prior to the study period. This study was approved by the ethics review board of Severance Hospital, and the requirement to obtain informed consent was waived owing to the retrospective study design (IRB approval number: 4-2019-0177).

### Identification of SNV Cases With Stroke and Comorbidities

SNV patients with incident stroke were defined as those admitted to a hospital and newly registered with ICD-10 codes for stroke (I60-I64) in the HIRA database after the diagnosis of SNV (20, 21). The follow-up duration was defined as the index date of SNV diagnosis to the date of stroke occurrence in patients with stroke and until the last follow-up date for patients without stroke. Comorbidities for stroke searched included hypertension (I10-15), diabetes mellitus (E10-14), atrial fibrillation/flutter (I48), and dyslipidemia (E78) within 1 year of the index date of SNV diagnosis.

### Estimation of Stroke Incidence in Patients With AAV Using In-Hospital Data

For internal validation purposes, the incidence of stroke was estimated by reviewing the medical records of 193 patients first diagnosed with AAV in Severance Hospital between December 2000 and December 2018. All patients were classified into AAV subgroups according to the 2007 European Medicines Agency algorithm for AAV and the descriptions provided by the 2012 Chapel Hill Consensus Conference. Demographic data, including age, sex, body mass index, and ANCA serotypes, and laboratory data, as well as the diagnosis and stroke subtypes were investigated. Birmingham Vasculitis Activity Score (version 3) (BVAS) and Five Factor Score (2009) were calculated as previously described (22, 23).

### Statistical Analysis

Continuous and categorical variables are presented as mean ± standard deviation and frequencies (percentage), respectively, and were compared using Student’s t-test and the chi-square test. To compare the incidence of stroke between SNV patients and the general population, the standardized incidence ratio (SIR) adjusted by age was calculated using data from the 2006 Korean Center for Disease Control & Prevention Report for the general population (24). The risk of stroke among the disease subgroups of SNV was compared by calculating age- and sex- adjusted incidence rate ratio (IRR) and 95% confidence interval (CI) using Poisson regression analysis with an offset for person-years. The cumulative incidence of stroke was calculated using the Kaplan–Meier method, and differences among disease subgroups were determined by the log-rank test. Factors associated with the incidence of stroke were investigated using Cox’s regression model by including medication usage as time-dependent covariates. SAS Enterprise Guide version 9.4 (SAS Institute Inc., Cary, NC) was used for all statistical analyses, and a p-value < 0.05 was considered statistically significant.

## Results

### Baseline Characteristics of SNV Patients With and Without Stroke

**Figure 1:**
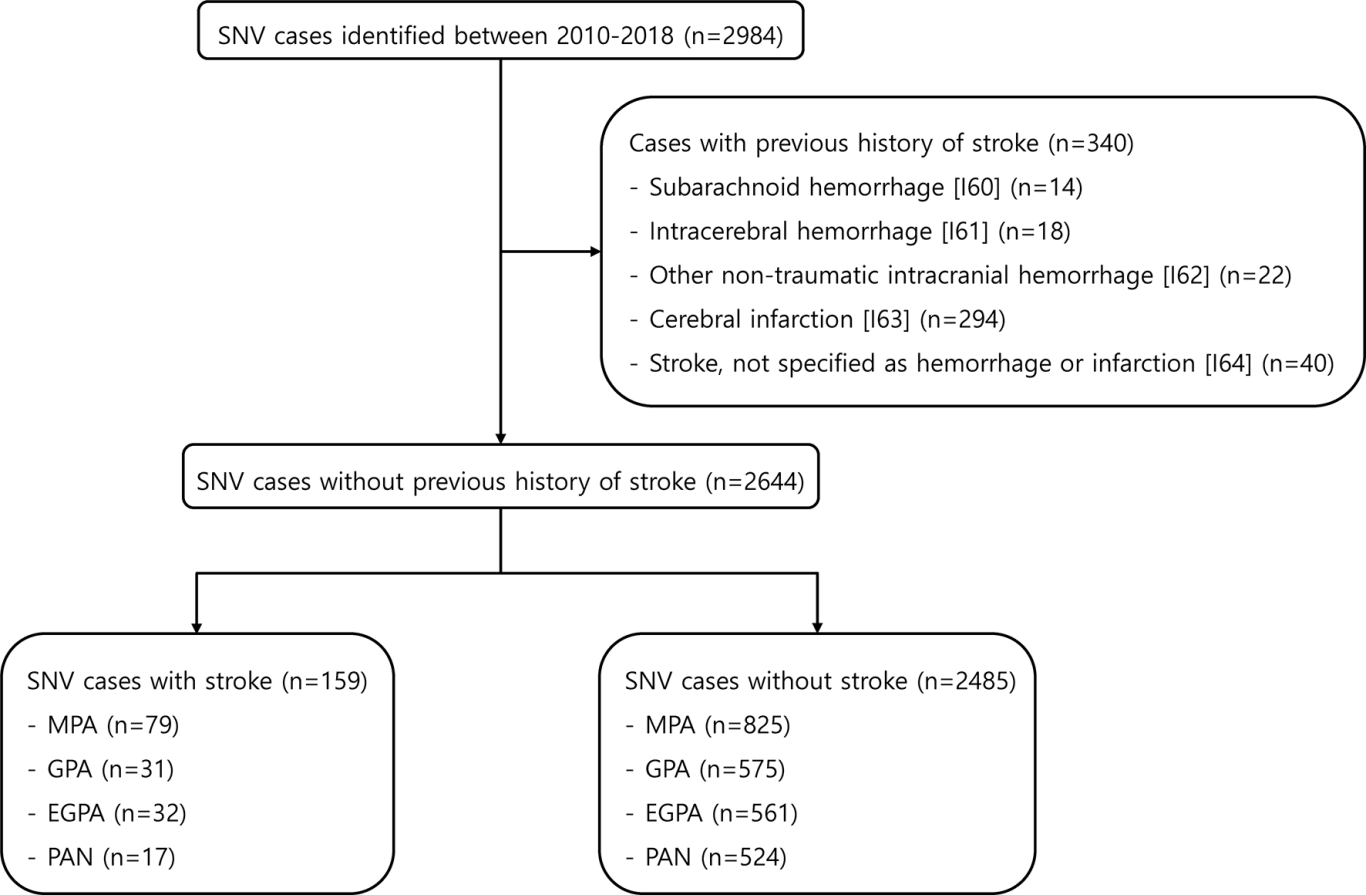
Selection of the study population from the HIRA database. HIRA, Health Insurance and Review Agency; SNV, systemic necrotizing vasculitis; MPA, microscopic polyangiitis; GPA, granulomatosis with polyangiitis; EGPA, eosinophilic granulomatosis with polyangiitis; PAN, polyarteritis nodosa.

A total of 2984 AAV and PAN cases were found in the HIRA database between 2010 and 2018. Of these, 340 patients who had been previously diagnosed with stoke (I60-I64) were excluded. In the remaining 2644, 159 (6.0%) patients were identified to have stroke after the diagnosis of SNV was established (**Figure 1**). The mean follow-up duration was 1.2 years for patients with stroke and 3.4 years for patients without stroke. When comparing the clinical characteristics between patients with stroke and those without, the age at diagnosis was higher (mean age 66.50 and 56.02, p<0.001) in patients with stroke. In addition, the diagnosis of MPA was more frequent in patients with stroke than in those without (49.7% vs. 33.2%, p<0.001). Regarding comorbidities, hypertension, diabetes mellitus, and dyslipidemia were more common in SNV patients with stroke, whereas the proportion of patients administered glucocorticoids ≥ 1 year, azathioprine/mizoribine, methotrexate, and statins was significantly higher in patients without stroke (**Table 1**). Significant differences were present regarding baseline clinical characteristics of SNV subgroups. In particular, patients with MPA were older and the proportion of patients with comorbidities at baseline was higher in MPA compared to other subgroups of SNV (**Supplementary Table 1**).

**Table 1:** Baseline clinical characteristics of SNV patients with stroke and those without stroke.

	Total, n=2644	Patients with stroke, n=159	Patients without stroke, n=2485	p-value
Age at diagnosis (years)	56.65 ± 16.84	66.50 ± 13.34	56.02 ± 16.85	<0.001
≤54	1052 (39.8)	28 (17.6)	1024 (41.2)	<0.001
55-74	1225 (46.3)	84 (52.8)	1141 (45.9)	
≥75	367 (13.9)	47 (29.6)	320 (12.9)	
Sex, n (%)				0.885
Male	1191 (45.1)	73 (45.9)	1118 (45.0)	
Female	1453 (54.9)	86 (54.1)	1367 (55.0)	
Diagnosis [ICD-10 code], n (%)				<0.001
MPA (M31.7)	904 (34.2)	79 (49.7)	825 (33.2)	
GPA (M31.3)	606 (22.9)	31 (19.5)	575 (23.1)	
EGPA (M30.1)	593 (22.4)	32 (20.1)	561 (22.6)	
PAN (M30.0)	541 (20.5)	17 (10.7)	524 (21.1)	
Geographic Area, n (%)				0.219
Seoul	1197 (45.3)	64 (40.3)	1133 (45.6)	
Outside Seoul	1447 (54.7)	95 (59.7)	1352 (54.4)	
Comorbidities [ICD-10 code]				
Hypertension [I10-15]				<0.001
No	1477 (55.9)	60 (37.7)	1417 (57.0)	
Yes	1167 (44.1)	99 (62.3)	1068 (43.0)	
Diabetes mellitus [E10-14]				<0.001
No	1813 (68.6)	87 (54.7)	1726 (69.5)	
Yes	831 (31.4)	72 (45.3)	759 (30.5)	
Atrial fibrillation/flutter [I48]				0.299
No	2576 (97.4)	153 (96.2)	2423 (97.5)	
Yes	68 (2.6)	6 (3.8)	62 (2.5)	
Dyslipidemia [E78]				0.005
No	1409 (53.3)	67 (42.1)	1342 (54.0)	
Yes	1235 (46.7)	92 (57.9)	1143 (46.0)	
Medication usage, n (%)				
Immunosuppressive agents				
Glucocorticoid usage ≥ 1 year	1225 (46.3)	31 (19.5)	1194 (48.1)	<0.001
Cyclophosphamide	1124 (42.5)	62 (39.0)	1062 (42.7)	0.399
Rituximab	252 (9.5)	8 (5.0)	244 (9.8)	0.064
Azathioprine/mizoribine	991 (37.5)	29 (18.2)	962 (38.7)	<0.001
Methotrexate	431 (16.3)	8 (5.0)	423 (17.0)	<0.001
Antiplatelet agents				
Aspirin	608 (23.0)	32 (20.1)	576 (23.2)	0.430
Clopidogrel	232 (8.8)	10 (6.3)	222 (8.9)	0.318
Statins	996 (37.7)	45 (28.3)	951 (38.3)	0.015

Values are expressed as mean (standard deviation) or number (percentages).

SNV, systemic necrotizing vasculitis; ICD, International classification of diseases; MPA, microscopic polyangiitis; GPA, granulomatosis with polyangiitis; EGPA, eosinophilic granulomatosis with polyangiitis; PAN, polyarteritis nodosa.

### Risk of Stroke in SNV Patients Compared to the General Population

**Figure 2:**
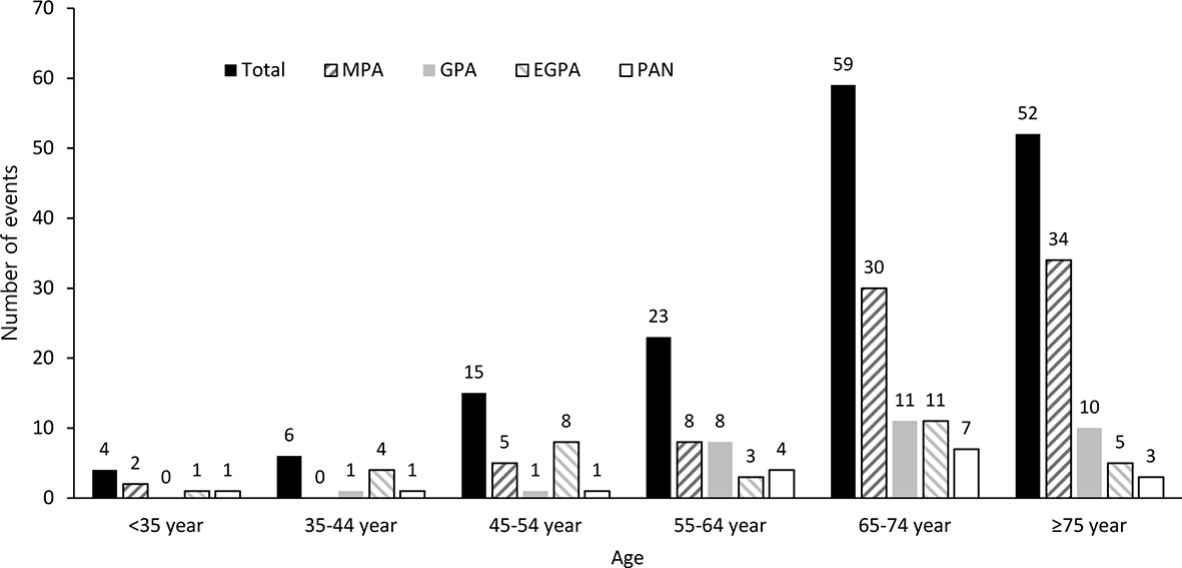
Age distribution of SNV patients on stroke occurrence. The incidence of stroke events was estimated by dividing age into 10-year intervals. SNV, systemic necrotizing vasculitis; MPA, microscopic polyangiitis; GPA, granulomatosis with polyangiitis; EGPA, eosinophilic granulomatosis with polyangiitis; PAN, polyarteritis nodosa.

The age distribution of SNV patients when they experienced stroke is shown in **Figure 2**. The total number of stroke events increased with age, and the peak was observed at 65-74 years (n=59). In those aged ≥75 years, it was slightly lower than that in patients aged 65-74 years (n=52).

Compared to the general population, the overall risk of stroke was significantly higher in patients with SNV (SIR 8.42, 95% CI 7.16-9.84); this was found to be the same in MPA, GPA, EGPA, and PAN. The overall risk of stroke was highest in patients with MPA (SIR 16.26, 95% CI 12.87-20.26). Moreover, when patients were divided according to age, such as age ≤ 54 years, age 55-74 years, and age ≥ 75 years, the SIR for stroke was highest in patients aged ≤ 54 years; this decreased as the age of patients increased (**Supplementary Figure 2**).

### Type and Incidence of Stroke After SNV Diagnosis

Among the 159 patients with stroke, cerebral infarction [I63] (n=115) was the most common stroke subtype, accounting for 72.3% of patients. Furthermore, the incidence of stroke was highest in patients with MPA (n=79, 49.7%), followed by those with EGPA, GPA, and PAN. Regarding the time for stroke occurrence after SNV diagnosis, 107 (67.3%) patients developed stroke within 1 year of SNV diagnosis. Meanwhile, the incidence of stroke gradually decreased over time after SNV was diagnosed, showing a similar pattern in all disease subgroups (**Table 2**).

**Table 2:** Type and incidence of stroke events after SNV diagnosis.

Type of stroke	Total	MPA	GPA	EGPA	PAN
Subarachnoid hemorrhage [I60]	14 (8.8)	7 (8.9)	3 (9.7)	4 (12.5)	0 (0.0)
Intracerebral hemorrhage [I61]	21 (13.2)	11 (13.9)	2 (6.5)	6 (18.7)	2 (11.8)
Other non-traumatic intracranial hemorrhage [I62]	6 (3.8)	2 (2.5)	1 (3.2)	2 (6.3)	1 (5.9)
Cerebral infarction [I63]	115 (72.3)	57 (72.1)	25 (80.6)	20 (62.5)	13 (76.5)
Stroke, not specified as hemorrhage or infarction [I64]	3 (1.9)	2 (2.5)	0 (0.0)	0 (0.0)	1 (5.9)
Total	159 (100.0)	79 (100.0)	31 (100.0)	32 (100.0)	17 (100.0)
Time of stroke occurrence after SNV diagnosis					
<1 year	107 (67.3)	56 (70.9)	19 (61.3)	22 (68.8)	10 (58.8)
1-3 years	29 (18.2)	13 (16.5)	6 (19.4)	6 (18.8)	4 (23.5)
>3 years	23 (14.5)	10 (12.7)	6 (19.4)	4 (12.5)	3 (17.7)

Values are expressed in number (percentages).

SNV, systemic necrotizing vasculitis; MPA, microscopic polyangiitis; GPA, granulomatosis with polyangiitis; EGPA, eosinophilic granulomatosis with polyangiitis; PAN, polyarteritis nodosa.

**Figure 3:**
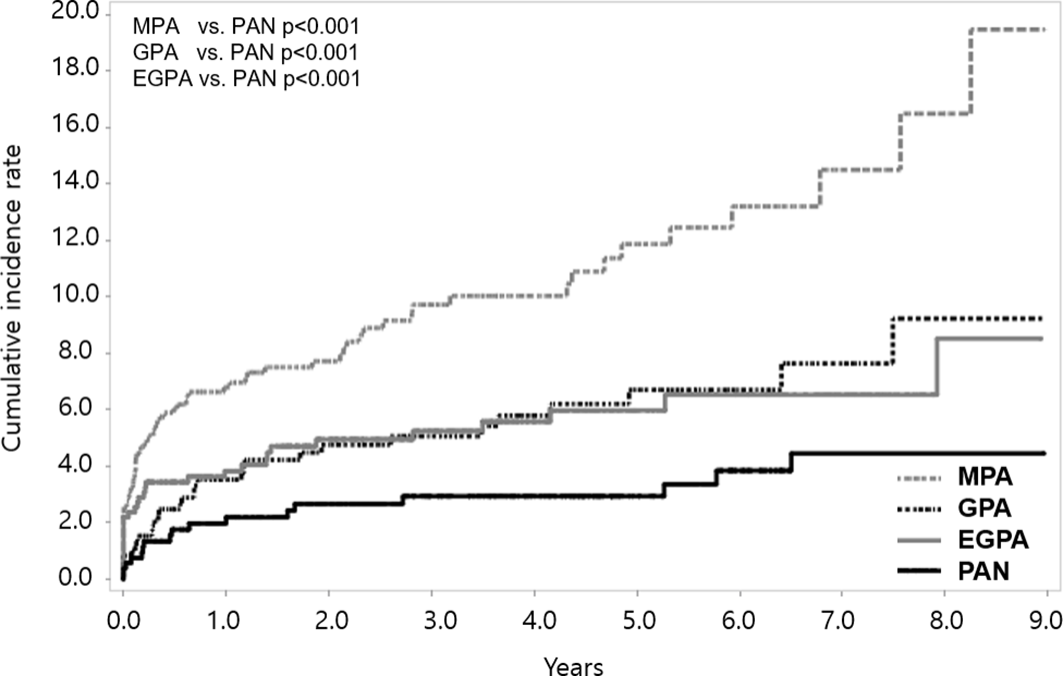
Cumulative incidence rate of stroke according to SNV subgroups. Among the SNV subgroups, the cumulative incidence rate of stroke was found to be highest in MPA. SNV, systemic necrotizing vasculitis; MPA, microscopic polyangiitis; PAN, polyarteritis nodosa; GPA, granulomatosis with polyangiitis; EGPA, eosinophilic granulomatosis with polyangiitis.

When we compared the cumulative incidence rate of stroke according to disease subgroups, patients with MPA, GPA, and EGPA had a higher incidence of stroke than those with PAN (all p<0.001). The cumulative incidence rate of stroke was highest in patients with MPA; it was 6.79 (95% CI 5.06-8.52), 10.02 (95% CI 7.65-12.39), and 19.46 (95% CI 11.56-27.36) at 1, 4, and 9 years, respectively (**Figure 3**). On comparing the incidence rates among the SNV subgroups, MPA patients exhibited the highest risk (crude IRR 4.85 vs. PAN, 95% CI 2.87-8.19); the risk of stroke was consistently higher in MPA patients than in PAN even after adjusting for age and sex (adjusted IRR 1.98, 95% CI 1.15-3.40) (**Supplementary Table 2**).

### Risk Factors Associated With the Occurrence of Stroke in SNV

In Cox’s hazard regression analysis, older age, the diagnosis of MPA, GPA, and EGPA, and comorbidities of hypertension, diabetes mellitus, and dyslipidemia were associated with increased risk of stroke in the unadjusted analysis. On the other hand, the administration of cyclophosphamide, azathioprine/mizoribine, and methotrexate were inversely associated with stroke occurrence. However, in an adjusted analysis, age at diagnosis [hazard ratio (HR) 1.05, 95% CI 1.03-1.06, p<0.001], the diagnosis of MPA (HR 1.88, 95% CI 1.08-3.26, p=0.025), and cyclophosphamide (HR 0.26, 95% CI 0.14-0.49, p<0.001), azathioprine/mizoribine (HR 0.34, 95% CI 0.18-0.65, p=0.001), methotrexate (HR 0.49, 95% CI 0.24-0.99, p=0.046), and statin (HR 0.50, 95% CI 0.32-0.80, p=0.004) administration were independent risk factors of stroke (**Table 3**).

**Table 3:** Factors associated with the risk of stroke in SNV patients.

	Crude hazard ratio			Adjusted hazard ratio		
	Hazard ratio	95% CI	p-value	Hazard ratio	95% CI	p-value
Age at diagnosis	1.06	(1.05-1.07)	<0.001	1.05	(1.03-1.06)	<0.001
Sex						
Male	1.08	(0.79-1.47)	0.646	0.99	(0.73-1.37)	0.977
Female	1.00 (ref)			1.00 (ref)		
Diagnosis						
MPA	3.69	(2.18-6.25)	<0.001	1.88	(1.08-3.26)	0.025
GPA	1.88	(1.04-3.41)	0.036	1.20	(0.65-2.19)	0.559
EGPA	1.89	(1.05-3.40)	0.035	1.25	(0.69-2.27)	0.460
PAN	1.00 (ref)			1.00 (ref)		
Geographic area						
Seoul	1.00 (ref)			1.00 (ref)		
Outside Seoul	1.26	(0.92-1.73)	0.149	1.25	(0.90-1.72)	0.179
Comorbidities						
Hypertension						
No	1.00 (ref)			1.00 (ref)		
Yes	2.42	(1.76-3.34)	<0.001	1.30	(0.91-1.87)	0.151
Diabetes mellitus						
No	1.00 (ref)			1.00 (ref)		
Yes	2.02	(1.48-2.76)	<0.001	1.26	(0.91-1.77)	0.170
Atrial fibrillation/flutter						
No	1.00 (ref)			1.00 (ref)		
Yes	1.59	(0.71-3.60)	0.263	0.95	(0.43-2.11)	0.898
Dyslipidemia						
No	1.00 (ref)			1.00 (ref)		
Yes	1.83	(1.33-2.51)	<0.001	1.29	(0.91-1.83)	0.149
Medication usage^†^						
Immunosuppressive agents						
Glucocorticoid usage ≥ 1 year	0.82	(0.47-1.41)	0.467	1.15	(0.65-2.02)	0.628
Cyclophosphamide	0.34	(0.18-0.63)	<0.001	0.26	(0.14-0.49)	<0.001
Rituximab	0.21	(0.01-3.35)	0.266	0.12	(0.01-1.97)	0.136
Azathioprine/mizoribine	0.36	(0.19-0.69)	0.002	0.34	(0.18-0.65)	0.001
Methotrexate	0.40	(0.20-0.82)	0.012	0.49	(0.24-0.99)	0.046
Antiplatelet agents						
Aspirin	1.33	(0.82-2.14)	0.249	1.20	(0.73-1.98)	0.480
Clopidogrel	1.56	(0.73-3.34)	0.250	1.12	(0.52-2.42)	0.767
Statins	0.77	(0.50-1.19)	0.243	0.50	(0.32-0.80)	0.004

^†^Medication usage was selected as time-dependent covariates.

SNV, systemic necrotizing vasculitis; CI, confidence interval; MPA, microscopic polyangiitis; GPA, granulomatosis with polyangiitis; EGPA, eosinophilic granulomatosis with polyangiitis; PAN, polyarteritis nodosa.

### Clinical Characteristics of AAV Patients With Stroke in the Hospital Database

We reviewed the medical records of patients diagnosed with AAV in the hospital and who experienced stroke. Among the 193 patients included, 12 (6.2%) experienced stroke during the follow-up. The baseline age and BVAS at diagnosis among patients with stroke were 64.5 years and 15.6, respectively, and the follow-up duration was 18.7 months. MPA (58.3%) was the most common diagnosis, followed by GPA (33.3%) and EGPA (8.3%). The disease duration after AAV diagnosis was less than 1 year in 9 (75.0%) patients, and ischemic subtype (91.7%) accounted for the majority of stroke events (**Table 4**).

**Table 4:** Baseline characteristics of AAV patients with stroke using in-hospital data.

Patient number	Age at initial diagnosis	Sex	BMI, kg/m2	BVAS	FFS (2009)	ANCA serotypes	ESR, mm/h	CRP, mg/L	Total cholesterol, mg/dL	Diagnosis	AAV disease duration (months)	Subtype of stroke
# 1	75	Male	26.0	24	3	ANCA (-)	90	95.8	150	MPA	1.0	Ischemic
# 2	56	Female	23.1	18	2	MPO-ANCA or p-ANCA (+)	120	94.3	165	MPA	0.0	Ischemic
# 3	76	Female	21.6	19	3	MPO-ANCA or p-ANCA (+)	116	178.7	119	MPA	4.0	Ischemic
# 4	73	Female	24.0	18	2	MPO-ANCA or p-ANCA (+)	110	216.5	113	GPA	0.0	Ischemic
# 5	77	Female	17.1	24	3	MPO-ANCA or p-ANCA (+)	120	137.5	152	MPA	1.0	Ischemic
# 6	57	Female	17.0	22	2	MPO-ANCA or p-ANCA (+)	62	146	134	MPA	1.0	Ischemic
# 7	69	Female	25.2	19	3	MPO-ANCA or p-ANCA (+)	94	83.4	99	MPA	46.0	Ischemic
# 8	71	Female	23.4	12	1	MPO-ANCA or p-ANCA (+)	61	100	146	GPA	4.0	Ischemic
# 9	75	Female	21.1	6	3	MPO-ANCA or p-ANCA (+)	90	109	117	GPA	0.0	Ischemic
# 10	31	Male	20.2	3	1	MPO-ANCA or p-ANCA (+)	48	12.03	202	MPA	0.0	Ischemic
# 11	60	Female	21.4	7	0	ANCA (-)	6	1	269	EGPA	109.0	Hemorrhage
# 12	54	Male	23.0	15	2	ANCA (-)	25	12.8	308	GPA	58.0	Ischemic

AAV, anti-neutrophil cytoplasmic antibody-associated vasculitis; BMI, body mass index; BVAS, Birmingham vasculitis activity score; FFS, five factor score; ANCA, anti-neutrophil cytoplasmic antibody; ESR, erythrocyte sedimentation rate; CRP, C-reactive protein; MPA, microscopic polyangiitis; MPO, myeloperoxidase; P, perinuclear; GPA, granulomatosis with polyangiitis; EGPA, eosinophilic granulomatosis with polyangiitis.

## Discussion

Even though the incidence of stroke is increased in patients with autoimmune rheumatic diseases (12), in patients with SNV, the incidence is unclear. In this study, using a nationwide claims database, we showed that a considerable number of patients with SNV (6.0%) are affected with stroke after disease diagnosis and the risk of stroke was significantly higher than that among the general population (SIR 8.42). Most stroke events in patients with SNV presented as ischemic subtypes, similar to the finding in the general population. In addition, among the disease subgroups, patients with MPA were most commonly affected with stroke. Importantly, these findings were reproduced through the in-hospital data, which revealed comparable results. Finally, the administration of immunosuppressive agents and statins showed clinical benefits in the prevention of stroke.

The risk of stroke is increased in autoimmune rheumatic diseases, such as rheumatoid arthritis and systemic lupus erythematosus (25). Likewise, the risk of stroke was significantly higher in SNV patients than in the general population. Even though the exact cause of this finding is unclear, the elevated risk of stroke may be attributable to the development of premature atherosclerosis in chronic inflammatory diseases (26). Otherwise, the acceleration of atherosclerosis can be also facilitated by direct vascular involvement in inflammation, which is mediated by antibodies or immune complexes (27). Additionally, the overproduction of proinflammatory cytokines, chemokines, and coagulation proteins may promote endothelial dysfunction and activation, resulting in atherosclerosis promotion (28). Finally, the activation of macrophages in autoimmune rheumatic diseases, which plays an important role in the progression of atherosclerotic lesions and the formation of foam cells, could be relevant to increased risk of stroke in SNV (29).

Age is an important factor contributing to stroke in the general population (9). According to the 2006 data from the Korean Center for Disease Control & Prevention Report, the incidence of stroke increases with age in the general population (21). Consistently, the baseline age of SNV patients with stroke was 66.5 years, which was approximately 10 years higher than that of SNV patients without stroke. Moreover, in patients with SNV, the incidence of stroke was much higher than that of the normal population of the same age group, implying that age and disease itself are independent risk factors of stroke. Collectively, it could be suggested that careful observation for the occurrence of stroke and the implementation of preventive measures of stroke are required in patients with SNV.

Several studies have investigated the risk of stroke in patients with SNV, but with inconsistent results. Although Mourguet et al. reported that patients with GPA and MPA experienced ischemic stroke four times more frequently than the general population, the observations by Berti et al. revealed that stroke incidence was eight times higher in patients with AAV than in the general population (30, 31). On the contrary, the risk of stroke was not apparent in patients with GPA (32). The discordant results could be related to the difference in disease subgroups analyzed, the number of patients included, and the type of stroke investigated. The results from our study are noteworthy as we observed the increased risk of stroke in SNV using a largest dataset, and the incidence of stroke in Asia has not been described in the literature. Nevertheless, given that geographic variations are present regarding the epidemiology of vasculitis and that racial and ethnic disparity should be considered in the incidence of stroke (33, 34), further research is necessary to validate the findings from our study.

Intriguingly, the observations from our study have demonstrated that 67.3% of patients have experienced stroke within 1 year of diagnosis, and the incidence of stroke gradually decrease during the disease course, which was found to be similar to the data from the United Kingdom (35). In addition, in a time-dependent Cox’s hazards regression analysis, treatment with immunosuppressive agents of cyclophosphamide, azathioprine/mizoribine, and methotrexate was inversely correlated with the occurrence of stroke. Of note, even though statistical significance was not demonstrated, the adjusted HR of developing stroke in patients treated with rituximab, which is now considered as a first line therapy for AAV, was found to be remarkably low. Owing to the fact that patients generally have the highest disease activity on initial presentation, and the reason to prescribe immunosuppressive drugs are used to maintain adequate disease control, it could be suggested that tight disease control is essential to reduce the occurrence of stroke in patients with SNV. This assumption could be supported by the analysis of in-hospital data, which showed that patients who developed stroke in the early disease phase generally had a higher disease activity (BVAS) and inflammatory markers of erythrocyte sedimentation rate (ESR) and C-reactive protein (CRP). Nonetheless, given that patients with SNV are usually followed-up more shortly after initial diagnosis, a possibility of detection bias from closer follow-up could not be excluded.

Among the disease subgroups, the incidence of stroke was the highest in MPA and lowest in PAN, and Cox’s hazards regression analysis revealed that the risk of stroke was increased in MPA compared to PAN. Although the direct cause of the difference of stroke according to disease subgroups is unknown, previous reports have described that stroke is known to be a relatively rare complication of PAN (36). Moreover, the higher incidence of stroke, especially in MPA, could be associated with the fact that MPA is an aggressive disease with severe renal and pulmonary symptoms and has a poor prognosis compared to other SNV subtypes (37, 38). Accordingly, considering the high inflammatory burden in MPA, it could be speculated that the occurrence of stroke is higher in MPA than in other disease subtypes. Meanwhile, it is reported that patients with MPA in South Korea and Japan most often manifest with renal involvement (39, 40), and a characteristic pathologic lesion in renal biopsy in MPA is rapidly progressive glomerulonephritis (5, 41). Based on the fact that impaired renal function is also a potential risk factor of stroke, the higher incidence of renal involvement in MPA could be associated with the increased risk of stroke (42).

Comorbidities that could influence in the occurrence of stroke include hypertension, diabetes mellitus, dyslipidemia, and atrial fibrillation (24). In this study, no significant association between the comorbidities investigated and stroke was observed in Cox’s hazards regression analysis. Surprisingly, we found that the use of statins was associated with reduced risk of stroke. Several different effects of statins could be considered in attenuating the risk of stroke in SNV. First, statins are effective in preventing atherosclerosis by reducing circulating low-density lipoproteins, and the attenuation of atherosclerotic lesions by statins could lead to a decreased incidence of stroke (43). Second, statins are able to provide non-lipid dependent vascular protective effects. Statins could maintain endothelial function through upregulation of endothelial nitric oxide synthase or antioxidant defense systems (44). Furthermore, statin inhibits recruitment, adhesion, and migration of inflammatory cells to help stabilize the inflamed vasculature (45). Third, statins could also provide immunomodulatory effects by suppressing T cell activation, which plays a crucial role in vascular inflammatory disorders (46). Statins could act as inhibitors of MHC-II-mediated T-cell activation by influencing MHC-II expression through IFN-γ and suppress Th1 responses (47). Furthermore, it was also shown that statins affect Th17 cell differentiation and directly inhibit IL-17 production in CD4+ cells (48). Finally, it was shown that treatment with statin could hamper macrophage activation, as well as reducing the expression of inflammatory cytokines and chemokines (45, 49). Therefore, the preventive effect of statins in stroke could be also mediated by controlling vascular dysfunction and providing immunomodulatory effects.

Although this is the first study to evaluate the risk of stroke in patients with SNV using a nationwide database, several drawbacks are present in this study. First, due to the inherent limitations of the HIRA data, we failed to analyze the effect of common modifiable risk factors for stroke, such as smoking, alcohol, and body mass index. Second, besides age, sex, and medication usage, other clinical data, such as disease activity and organ involvement, were not identifiable through the HIRA database. In addition, laboratory data regarding cholesterol profiles and its changes, as well as ANCA serotypes and inflammatory markers of ESR and CRP, were also not accessible. Third, even though a Cox’s hazard regression analysis was performed to identify risk factors associated with stroke in SNV, large disparities of baseline characteristics were present between the disease subgroups. Fourth, the risk of stroke in SNV compared to the general population was assessed using a nationwide report as a reference, and the comorbidities of patients were investigated only by using the ICD-10 codes. Thus, the relationship between comorbidities in SNV and stroke should be better verified in future studies. Fifth, in this study, both the occurrence of ischemic and hemorrhagic stroke was analyzed as stroke event. However, as the underlying pathogenesis of ischemic and hemorrhagic stroke is different, the precise mechanism leading to increased stroke in SNV remains to be further investigated. Finally, owing to the relatively small number of patients with stroke and those treated with rituximab, the effect of rituximab therapy in reducing stroke might not have reached statistical significance.

## Conclusion

In conclusion, the results of our study demonstrated that the overall incidence of stroke is elevated in patients with SNV, especially in the early phase of disease diagnosis. In addition, differences were found regarding the incidence of stroke according to disease subgroups. Moreover, the use of immunosuppressive agents and statins was associated with decreased risk of stroke. Our results indicate that special attention should be given regarding the incidence of stroke in patients with SNV, and adequate disease control and use of statins could be beneficial in minimizing the risk of stroke.

## Data Availability Statement

The raw data supporting the conclusions of this article will be made available by the authors, without undue reservation.

## Ethics Statement

The studies involving human participants were reviewed and approved by Ethics review board of Severance Hospital. Written informed consent for participation was not required for this study in accordance with the national legislation and the institutional requirements.

## Author Contributions

SA and S-WL designed the report and wrote the paper. SA, MH, and JY participated in data acquisition and interpretation. SA, Y-BP, and S-WL drafted and revised the manuscript. SA, JY, and S-WL designed the concept and approved the final paper. All authors contributed to the article and approved the submitted version.

## Funding

This work was supported by the Research Program funded by the Korea Centers for Disease Control and Prevention (2019-ER6904-00), a grant from the Korea Health Technology R&D Project through the Korea Health Industry Development Institute, funded by the Ministry of Health and Welfare, Republic of Korea (HI14C1324), and a faculty research grant of Yonsei University College of Medicine (6-2019-0184). The funders had no role in study design, data collection and analysis, decision to publish, or preparation of the manuscript.

## Conflict of Interest

The authors declare that the research was conducted in the absence of any commercial or financial relationships that could be construed as a potential conflict of interest.
