# Supplementary material for: Risk of Stroke in Systemic Necrotizing Vasculitis: A Nationwide Study Using the National Claims Database
Source: Front Immunol. 2021 Mar 31;12:629902. doi: 10.3389/fimmu.2021.629902 (PMC8046646; doi:10.3389/fimmu.2021.629902)
Supplement: Supplementary file 1 [file DataSheet_1.pdf]

Supplementary Materials

Supplementary Table 1. Comparison of baseline clinical characteristics of SNV subgroups

	MPA (n=904)	GPA (n=606)	EGPA (n=593)	PAN (n=541)	p-value
Age at diagnosis (years)	64.26±14.00	58.30±15.06	53.01±15.57	46.08±17.72	<0.001
≤54	161 (17.8)	215 (35.5)	314 (53.0)	362 (66.9)	<0.001
55-74	533 (59.0)	304 (50.2)	237 (40.0)	151 (27.9)	
≥75	210 (23.2)	87 (14.3)	42 (7.0)	28 (5.2)	
Sex, n (%)					
Male	375 (41.5)	291 (48.0)	282 (47.5)	243 (44.9)	0.040
Female	529 (58.5)	315 (52.0)	311 (52.5)	298 (55.1)	
Geographic Area, n (%)					
Seoul	456 (50.4)	237 (39.1)	232 (39.1)	272 (50.3)	<0.001
Outside Seoul	448 (49.6)	369 (60.9)	361 (60.9)	269 (49.7)	
Comorbidities [ICD-10 code]					
Hypertension [I10-15]					
No	377 (41.7)	347 (57.3)	381 (64.3)	372 (68.8)	<0.001
Yes	527 (58.3)	259 (42.7)	212 (35.7)	169 (31.2)	
Diabetes mellitus [E10-14]					
No	547 (60.5)	405 (66.8)	438 (73.9)	423 (78.2)	<0.001
Yes	357 (39.5)	201 (33.2)	155 (26.1)	118 (21.8)	
Atrial fibrillation/flutter [I48]					
No	867 (95.9)	593 (97.9)	581 (98.0)	535 (98.9)	0.003
Yes	37 (4.1)	13 (2.1)	12 (2.0)	6 (1.1)	
Dyslipidemia [E78]					
No	384 (42.5)	318 (52.5)	344 (58.0)	363 (67.1)	<0.001
Yes	520 (47.5)	288 (47.5)	249 (42.0)	178 (32.9)	
Medication usage, n (%)					
Immunosuppressive agents					
Glucocorticoid usage ≥ 1year	377 (41.7)	302 (49.8)	336 (56.7)	210 (38.8)	<0.001
Cyclophosphamide	503 (55.6)	312 (51.5)	194 (32.7)	115 (21.3)	<0.001
Rituximab	158 (17.5)	86 (14.2)	3 (0.5)	5 (0.9)	<0.001
Azathioprine/mizoribine	392 (43.4)	243 (40.1)	183 (30.9)	173 (32.0)	<0.001
Methotrexate	61 (6.8)	175 (28.9)	74 (12.5)	121 (22.4)	<0.001

Antiplatelet agents					
Aspirin	230 (25.4)	132 (21.8)	98 (16.5)	148 (27.4)	<0.001
Clopidogrel	106 (11.7)	38 (6.3)	41 (6.9)	47 (8.7)	<0.001
Statins	397 (43.9)	257 (42.4)	194 (32.7)	148 (27.4)	<0.001

Values are expressed as mean (standard deviation) or number (percentages).

SNV: Systemic necrotizing vasculitis, ICD: International classification of diseases, MPA: Microscopic polyangiitis, GPA: Granulomatosis with polyangiitis, EGPA: Eosinophilic granulomatosis with polyangiitis, PAN: Polyarteritis nodosa

Supplementary Table 2. Estimation of incidence rate ratio of stroke among the SNV subgroups

Diagnosis	# of events	Person-years	Incidence rate (/1000 person-years)	95% CI	Crude incidence rate ratio	95% CI	Adjusted incidence rate ratio*	95% CI
MPA	79	2249.29	35.12	(27.94-43.45)	4.85	(2.87-8.19)	1.98	(1.15-3.40)
GPA	31	1992.43	15.56	(10.70-21.70)	2.15	(1.19-3.88)	1.14	(0.63-2.08)
EGPA	32	2149.22	14.89	(10.31-20.66)	2.06	(1.14-3.70)	1.44	(0.80-2.59)
PAN	17	2348.47	7.24	(4.32-11.24)	1.00 (ref)		1.00 (ref)	
Total	159	8739.41	18.19	(15.51-21.17)				

*Adjustment was performed by age and sex.

SNV: Systemic necrotizing vasculitis, CI: Confidence interval, MPA: Microscopic polyangiitis, GPA: Granulomatosis with polyangiitis, EGPA: Eosinophilic granulomatosis with polyangiitis, PAN: Polyarteritis nodosa

Supplementary Figure 1. Generation of the HIRA database and data utilization

A schematic figure representing the generation and utilization of HIRA data. The data of healthcare utilization of a patient, which is submitted by the hospitals to the HIRA for financial reimbursement after patient care, are accumulated and is stored in the data warehouse. These data are transmitted to the NHIS and monetary reimbursement is made to the hospitals when it conforms to the insurance standards; researchers are authorized to assess the HIRA database through a remote account when the study is both approved by the HIRA and the medical institution where the researcher is affiliated.

HIRA: Health Insurance and Review Agency, NHIS: National Health Insurance System

Supplementary Figure 2. Overall and disease-subgroup specific risk of stroke in SNV patients

The overall and SNV subgroup-specific SIR of stroke adjusted by age was calculated.

SNV: Systemic necrotizing vasculitis, SIR: Standardized incidence ratio, CI: Confidence interval, MPA: Microscopic polyangiitis, GPA: Granulomatosis with polyangiitis, EGPA: Eosinophilic granulomatosis with polyangiitis, PAN: Polyarteritis nodosa
