# Supplementary figures and images for: Risk of Stroke in Systemic Necrotizing Vasculitis: A Nationwide Study Using the National Claims Database
Source: Front Immunol. 2021 Mar 31;12:629902. doi: 10.3389/fimmu.2021.629902 (PMC8046646; doi:10.3389/fimmu.2021.629902)

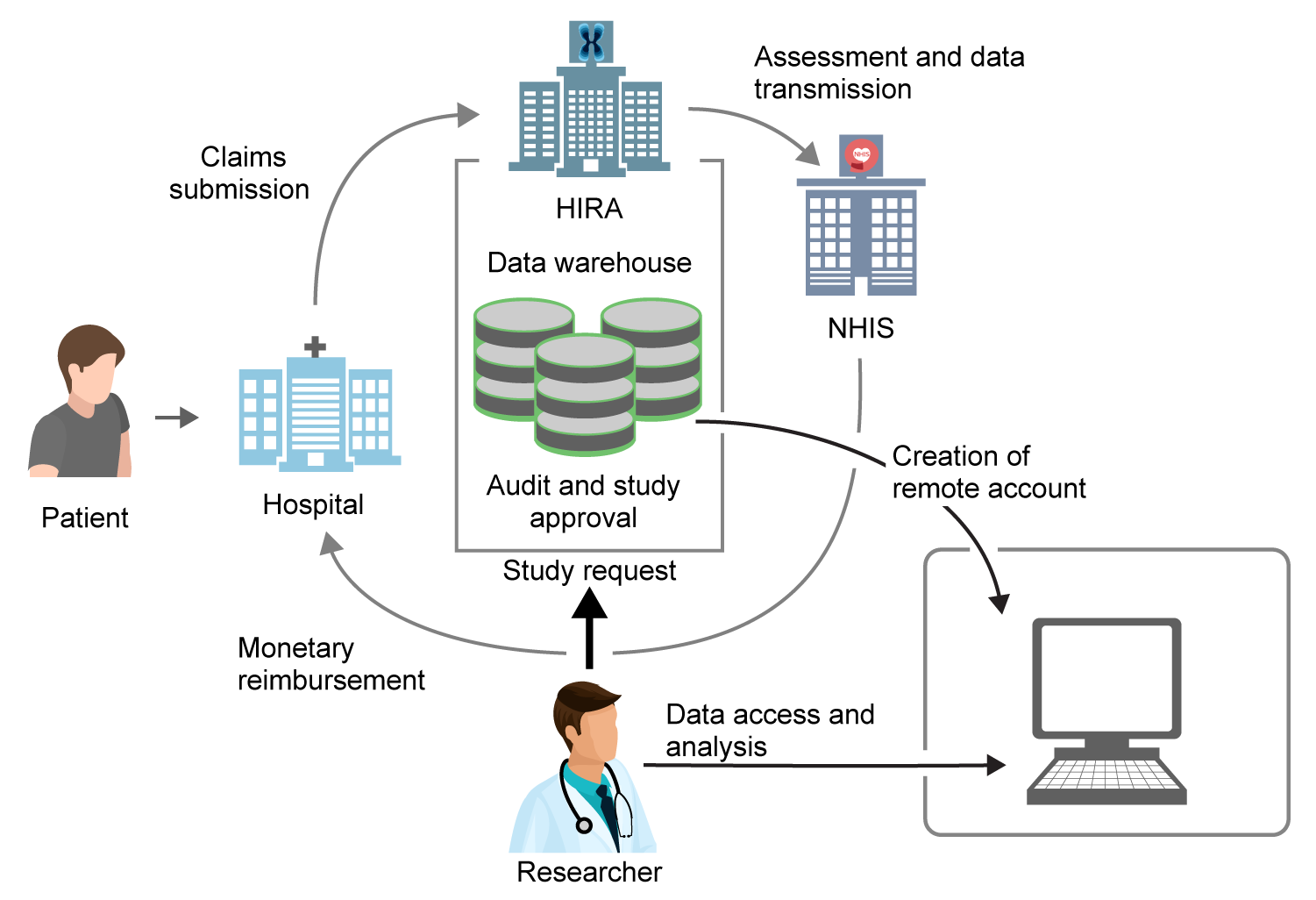

Supplement: Supplementary file 2 [file Image_1.tif]

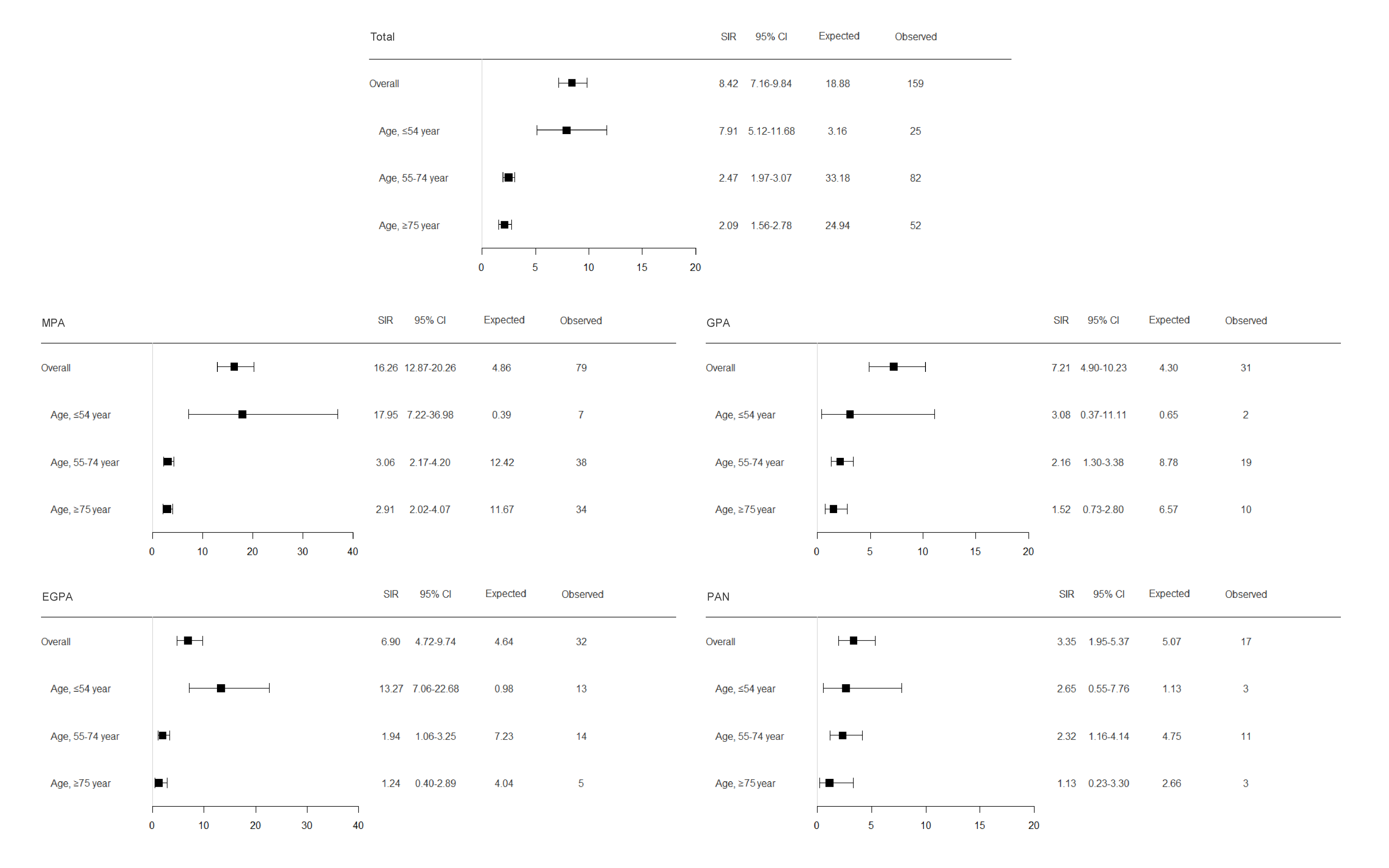

Supplement: Supplementary file 3 [file Image_2.tif]
